# Diverse Roles of Axonemal Dyneins in *Drosophila* Auditory Neuron Function and Mechanical Amplification in Hearing

**DOI:** 10.1038/srep17085

**Published:** 2015-11-26

**Authors:** Somdatta Karak, Julie S. Jacobs, Maike Kittelmann, Christian Spalthoff, Radoslaw Katana, Elena Sivan-Loukianova, Michael A. Schon, Maurice J. Kernan, Daniel F. Eberl, Martin C. Göpfert

**Affiliations:** 1 Department of Cellular Neurobiology, University of Göttingen, 37077 Göttingen, Germany; 2 Biology Department, University of Iowa, Iowa City, IA 52242; 3 Department of Neurobiology and Behavior and Center for Developmental Genetics, Stony Brook University, Stony Brook, NY 11794.

## Abstract

Much like vertebrate hair cells, the chordotonal sensory neurons that mediate hearing in *Drosophila* are motile and amplify the mechanical input of the ear. Because the neurons bear mechanosensory primary cilia whose microtubule axonemes display dynein arms, we hypothesized that their motility is powered by dyneins. Here, we describe two axonemal dynein proteins that are required for *Drosophila* auditory neuron function, localize to their primary cilia, and differently contribute to mechanical amplification in hearing. Promoter fusions revealed that the two axonemal dynein genes Dm*dnah3* (=*CG17150*) and Dm*dnai2* (=*CG6053*) are expressed in chordotonal neurons, including the auditory ones in the fly’s ear. Null alleles of both dyneins equally abolished electrical auditory neuron responses, yet whereas mutations in Dm*dnah3* facilitated mechanical amplification, amplification was abolished by mutations in Dm*dnai2*. Epistasis analysis revealed that Dm*dnah3* acts downstream of Nan-Iav channels in controlling the amplificatory gain. Dm*dnai2*, in addition to being required for amplification, was essential for outer dynein arms in auditory neuron cilia. This establishes diverse roles of axonemal dyneins in *Drosophila* auditory neuron function and links auditory neuron motility to primary cilia and axonemal dyneins. Mutant defects in sperm competition suggest that both dyneins also function in sperm motility.

Cilia can be categorized as motile or primary ones based on the structure of their microtubule axonemes^1^: Motile cilia display ‘9 + 2’ axonemes, consisting of nine circularly arranged microtubule doublets (‘9’) that surround a central pair of microtubules (‘+2’). Primary cilia, by contrast, present ‘9 + 0’ axonemes that lack the latter central microtubule pair. Primary cilia usually lack axonemal dynein arms and, accordingly, are immotile: instead of generating movements with dynein motors, these cilia serve as chemo- or mechanosensory organelles^1^. Nevertheless, motile primary cilia that bear axonemal dynein arms exist, such as the vertebrate nodal cilia that promote left-right asymmetry during development^2^,^3^. A second example might be found in *Drosophila*, whose auditory sensory neurons are motile^4^ and are endowed with primary cilia that present dynein arms^5^,^6^. The latter arms are confined to the proximal region of the auditory cilia^6^, and while they look like dynein arms in electron-micrographs, their molecular composition is unknown^7^,^8^.

The motility of *Drosophila* auditory neurons cannot be accessed directly but is betrayed by the fly’s auditory mechanics. Hearing in *Drosophila* is mediated by the antenna whose distal part vibrates in response to sound^8^. This vibration is directly coupled to the ca. 500 chordotonal mechanosensory neurons of Johnston’s organ in the antenna’s proximal part^8^. Each neuron bears one primary cilium, and the neurons actively amplify antennal vibrations on a cycle-by-cycle basis, documenting their motile properties^4^,^9^,^10^. Biophysically, the source of this amplification in hearing might reside in (i) the interplay between force-gated ion channels and associated motor protein, (ii) the collective behavior of cells or motor proteins, or (iii) ionically driven conformational changes of force-gated ion channels or other proteins, without the involvement of ATP-consuming motors^11^. Modelling studies revealed that the first scenario, the interplay between force-gated channels and motors, might be realized in the *Drosophila* ear^10^, and amplification was shown to require the NOMPC (=TRPN1) transient receptor potential (TRP) channel^12^,^13^, which localizes to the tips of auditory neuron cilia^14^,^15^ and is gated by force^16^,^17^. The gain of amplification is negatively controlled by the TRPV channel subunits Nan and Iav^12^, which form a heteromeric Nan-Iav channel complex in the proximal ciliary region that presents dynein arms^18^,^19^. The mere presence of these arms suggests that *Drosophila* auditory neurons might use axonemal dyneins to drive mechanical amplification, a possibility that seems supported by genetic evidence: both mechanical amplification and the dynein arms are disrupted by mutations in genes that are implicated in axonemal dynein arm assembly, including for example *fd3f* (Ref. 20), *tilB* (Refs 4,21), *zmynd10* (Refs 22,23), d*yx1c1* (Refs 22,24), and *hmw* (Ref. 25). Although these mutant defects suggest that axonemal dyneins might power mechanical amplification, genetic evidence demonstrating that this amplification involves axonemal dynein genes has hitherto not been reported^26^.

Axonemal dynein arms can be categorized into outer and inner ones that differ in their molecular composition: each arm represents a multi-protein complex that consists of several axonemal dynein heavy, intermediate, and light chain subunits^27^. The sequenced *Drosophila* genome includes eleven axonemal dynein heavy chain genes^28^,^29^, and twelve genes encoding WD-repeat, dynein intermediate chain proteins (Figs. S2,3). Some proteins of each type seem specific to the sperm flagellum: for instance, three of the axonemal heavy chain genes are on the Y chromosome and so are normally present only in males, and several subunits encoded on other chromosomes are expressed at high levels only in testis^30^. Here we describe two axonemal dynein subunits, an inner arm heavy chain and a WD-repeat intermediate chain, that are expressed in both males and females, in chordotonal sensory neurons including those of Johnston’s organ. Using mutations of these genes, we tested whether and, if so, how axonemal dyneins and ciliary motility contribute to *Drosophila* auditory neuron function and mechanical amplification in fly hearing.

## Results

### *Dm*DNAH3 is a monomeric dynein heavy chain

*CG17150* is one of eleven axonemal dynein heavy chain genes in *Drosophila*^28^,^29^ (Fig. S1). It falls into the IAD-3 (inner-arm dynein-3) subgroup, described as inner arm-associated and single-headed (monomeric), together with the *Drosophila Dhc62B*/*CG15804* and *Dhc36C/CG5526* and human *DNAH3* and *DNAH7* gene products^28^,^29^. Among the human dynein heavy chains, CG17150 is most similar (50% amino acid identity) to DNAH3, and we name the gene Dm*dnah3* for this orthology. The IAD-3 heavy chain subgroup has several representatives in each ciliated species, but information about their function is scant. A mutation of *Chlamydomonas* that deletes most of an IAD-3 heavy chain (DHC9) lacks a dynein inner arm and shows reduced flagellar beat frequency, especially in more viscous media, suggesting that this motor is important for motility under load^31^.

### *Dm*DNAI2 is an outer arm IC2 protein

*CG6053* encodes a WD-repeat, dynein intermediate chain (IC) (Fig. S1), and is one of twelve dynein IC genes in *Drosophila* (Figs S2–4). When compared to the dynein ICs in *Chlamydomonas*^29^, CG6053 protein is most similar to ODA6 (36.7% amino acid identity), placing it with human DNAI2 (44.7% identity) in the IC2 subgroup (Figs. S2–4), and we name it *Dm*DNAI2 for its human ortholog. In both *Chlamydomonas* flagella and human cilia, loss-of-function mutations in IC2 proteins prevent the assembly of the entire outer arm complex: mutant cilia lack outer arms and their constituent heavy chain proteins^32^,^33^, while partial *ODA6* revertants have specific effects on flagellar beating^34^. Structural labeling locates the protein near the base of the outer dynein arm, where it controls the activity of both the outer and inner arm^35^. IC2 proteins are thus conserved across ciliated eukaryotes, and required both for outer dynein arm assembly and for normal motility of the assembled axoneme.

Two other *Drosophila* IC proteins, encoded by *CG1571* and *CG10859,* also fall within the IC2 subclass, but are not as similar to their algal and human homologs as is *CG6053*/Dm*dnai2*. RNA-seq expression data for both genes^30^ show high transcript levels in testis and little or no expression in females, suggesting that they function in the sperm flagellum. In contrast, *CG6053* transcripts, though less abundant, are expressed and more broadly distributed in both males and females^30^, consistent with a function outside the male germline.

### Dm*dnah3* and Dm*dnai2* are expressed in chordotonal neurons

**Figure 1:**
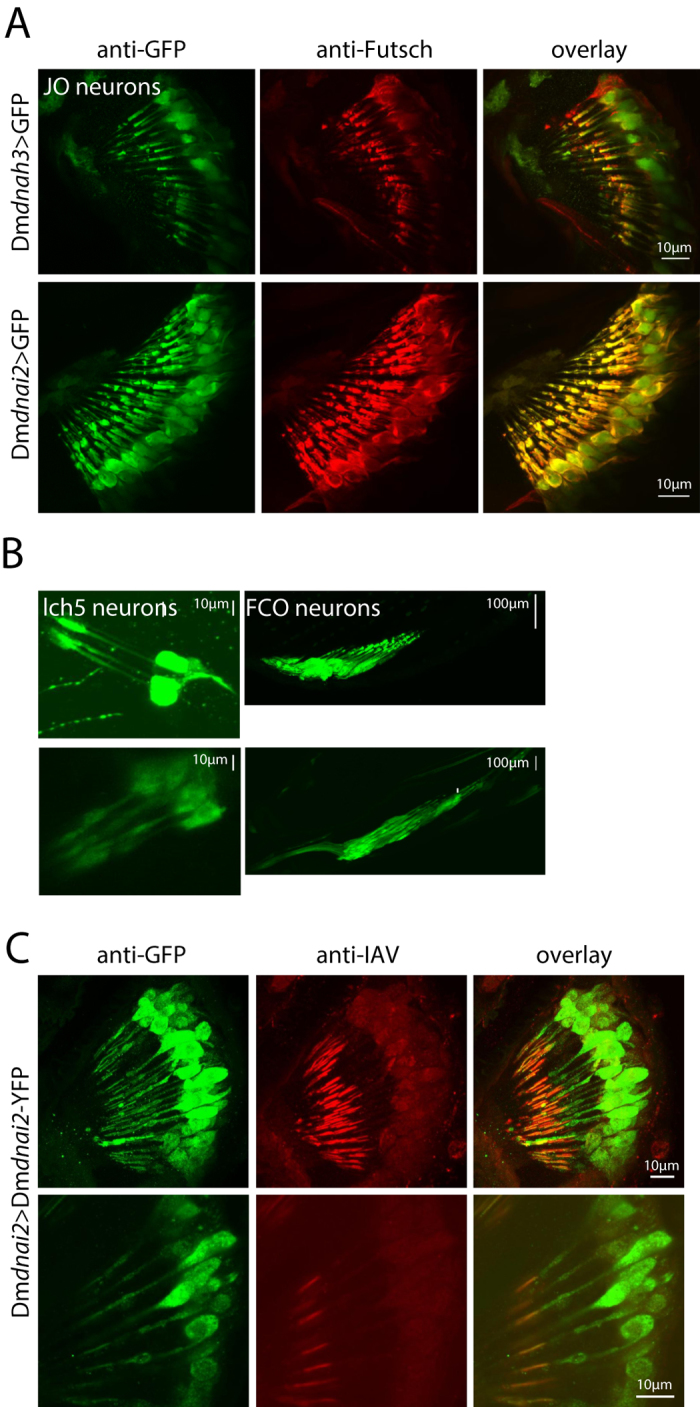
Axonemal dynein expression and localization in chordotonal neurons. (**A,B**) Expression of Dm*dnah3*-GAL4 and Dm*dnai2*-GAL4 in Johnston’s organ neurons (**A**) and in chordotonal neurons of the larval pentascolopidial organ (lch5) and the adult femoral chordotonal organ (FCO) (**B**). Expression was assessed by driving an UAS-GFP reporter via Dm*dnah3*-GAL4 or Dm*dnai2*-GAL4. GFP signals were enhanced with an anti-GFP antibody. Johnston’s organ neurons were counterstained with the neuronal antibody 22C10 (**A**). (**C**) *Dm*DNAI2 protein localization in Johnston’s organ neurons, revealed by expressing UAS-Dm*dnai2*-YFP under the control of Dm*dnai2*-Gal4. YFP signals were enhanced with an anti-GFP antibody and counterstained with an anti-Iav antibody, which recognizes Iav protein in the proximal region of the cilia. Within the cilia, *Dm*DNAI2-YFP signals superimpose with Iav in the proximal ciliary region but do not extend distally in the ciliary tips.

To characterize the cellular expression patterns of Dm*dnah3* and Dm*dnai2*, we generated fusions between the Dm*dnah3* or Dm*dnai2* enhancer/promoter regions and the yeast transcription activator GAL4 (see Supplementary Methods). To visualize GAL4 expression, transgenic flies expressing Dm*dnah3*-GAL4 and Dm*dnai2-*GAL4 were crossed to flies expressing a green fluorescent protein (GFP) under the control of upstream activating sequence (UAS) elements (UAS-GFP). GFP signals were enhanced with an anti-GFP antibody, and neurons were counterstained with the monoclonal anti-Futsch antibody 22c10 (Ref. 36). Labelling induced by the Dm*dnah3* and Dm*dnai2* enhancer/promoter regions was observed in Johnston’s organ, the chordotonal auditory sensory organ in the fly’s antenna (Fig. 1A). Within this organ, anti-GFP and 22c10 staining superimposed, documenting that virtually all its 500 sensory neurons express Dm*dnah3* and Dm*dnai2* (Fig. 1A). Apart from Johnston’s organ neurons, expression of Dm*dnah3* and Dm*dnai2* was also observed in other chordotonal neurons, including those of the femoral chordotonal organ (FCO) in the fly’s leg and those of the larval pentascolopidial organ (lch5) (Fig. 1B). No expression was seen in the central nervous system or ciliated chemoreceptors and mechanosensory bristle neurons whose cilia reportedly lack dynein arms^7^. Chordotonal sensory neurons thus seem to be the only *Drosophila* neurons that express Dm*dnah3* and Dm*dnai2.*

To gain insights into the subcellular localization of axonemal dyneins, we generated transgenic flies carrying a *U*AS-Dm*dnai2*-YFP construct, in which *Dm*DNAI2 is tagged with yellow fluorescent protein (YFP). Expression of *UAS-*Dm*dnai2-YFP* construct was targeted to Johnston’s organ neurons using Dm*dnai2*-GAL4, and the subcellular localization of *Dm*DNAI2-YFP protein was assessed after enhancing YFP fluorescence with an anti-GFP antibody that recognizes YFP. *Dm*DNAI2-YFP fluorescence was observed in Johnston’s organ neuron somata and dendrites (Fig. 1C). Within the dendrites, fluorescence signals extended into the mechanosensory cilia, where the signals were confined to the proximal ciliary region that harbors both Nan-Iav TRPV channels and dynein arms^6^. Counterstaining with an anti-Iav antibody confirmed that *Dm*DNAI2-YFP co-localizes with Iav in the proximal ciliary region but is absent from the distal tips of the cilia that lack Iav protein and dynein arms^6^. Hence, within *Drosophila* auditory neuron cilia, at least *Dm*DNAI2 occurs in that region that presents dynein arms.

### Mutations in Dm*dnah3* and Dm*dnai2* affect auditory neuron function and mechanical amplification in the ear

To determine whether axonemal dyneins are required for the motility of Johnston’s organ neurons, we next tested for mutant alterations in mechanical amplification. Flies carrying *Minos (Mi)* transposon insertions in Dm*dnah3* and Dm*dnai2* were used as mutants, i.e. *Mi*{*ET1*}*CG17150*^*MB05004*^ (hereafter named Dm*dnah3*^*1*^) and *Mi*{*ET1*}*CG6053*^*MB06262*^ (hereafter named Dm*dnai2*^*1*^). PCR confirmed the genomic positions of the two *Mi* insertions (Fig. S1), and both Dm*dnah3*^*1*^ and Dm*dnai2*^*1*^ seemed to be null mutations as no transcripts were detected by RT-PCR (Fig. S5). To assess mechanical amplification, we exposed the flies to pure tones at the individual mechanical best frequencies of their antennae and simultaneously monitored the resulting antennal displacement and electrical compound action potentials of Johnston’s organ neurons that were recorded extracellularly from the antennal nerve. Mechanical best frequencies of the antennae were deduced from power spectra of their mechanical free fluctuations in the absence of acoustic stimulation, and tone intensities were measured as the sound particle velocity at the position of the flies^12^.

**Figure 2:**
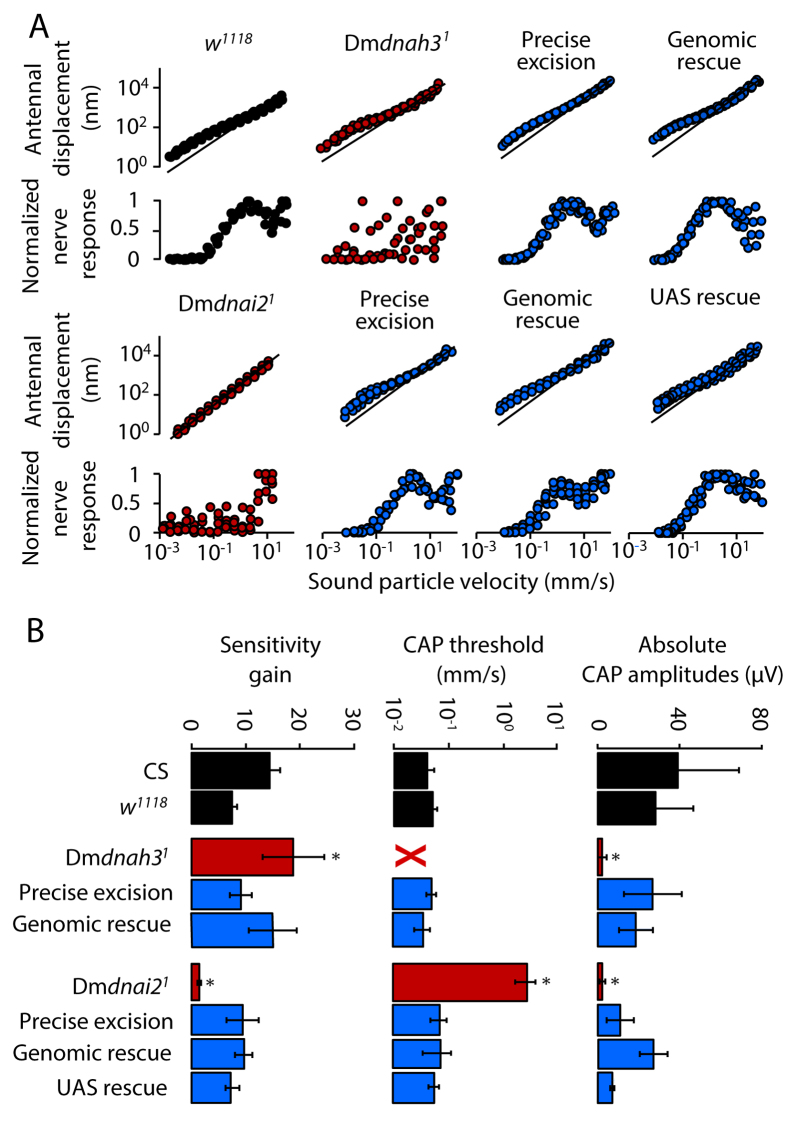
Effects of mutations in Dm*dnah3* and Dm*dnai2* on hearing. (**A**) Pure tone-evoked antennal displacement (top) and normalized amplitude of the associated nerve response (bottom) as a function of the sound particle velocity of the tone. Tone frequencies were adjusted to match the mechanical best frequency of the antenna^12^, and lines indicate linear antennal mechanics. In *w*^*1118*^ controls, the antenna’s displacement displays a compressive nonlinearity that aligns with the dynamic range of the nerve response and arises from mechanical amplification by JO neurons^12^ (data from N ≥ 5 flies/antennae per strain). (**B**) Corresponding sensitivity gain due to mechanical amplification, sound particle velocity thresholds of the nerve responses, and maximum amplitudes of the nerve responses extracted from the data in (A) (means ± SD). CS: Canton S wild-type flies. X: not accessible because the nerve response is entirely lost. *significant difference (p < 0.05) from *w*^*1118*^ controls: (Kruskal-Wallis test followed by two-tailed Mann-Whitney U-tests with Bonferroni correction). Nerve responses were measured as compound action potentials (CAP). Mechanical amplification is enhanced in Dm*dnah3*^*1*^ mutants, where the nerve response is largely abolished. Both the nerve response and normal amplification are restored by precise excision of the responsible Minos insertion and by a genomic rescue of Dm*dnah*^*3*^. Dm*dnai*^*2*^ mutants lack both the antennal nonlinearity and the nerve response, which are restored by excising the respective Mi insertion, genomic rescue, and by targeting the expression of UAS-Dm*dnai2*-YFP to Johnston’s organ neurons via the chordotonal neuron driver F-GAL4.

In *w*^*1118*^ genetic background controls, sound particle velocities exceeding ca. 50 μm/s elicited robust electrical compound responses that, increasing sigmoidally with the sound intensity, reached maximum potential amplitudes of ca. 40 μV (Fig. 2A,B). These electrical sound responses of Johnston’s organ neurons were virtually abolished in both Dm*dnah3*^*1*^ and Dm*dnai2*^*1*^ mutants, whereby the latter mutants retained some residual responses (maximum potential amplitudes around 2 μV) to intense sound stimuli (particle velocities >ca. 5 mm/s) (Fig. 2A,B).

The loss of sensitive electrical sound responses in Dm*dnai2*^*1*^ mutants was associated with a loss of mechanical amplification: in control flies, antennal displacements showed the characteristic nonlinear intensity scaling (Fig. 2A) that, arising from motile responses of Johnston’s organ neurons^10^,^12^, amplified the ear’s mechanical input with a gain of approximately 10 (Fig. 2B). This mechanical amplification was completely abolished in Dm*dnai2*^*1*^ mutants (Fig. 2A), as witnessed by amplificatory gains of around one (Fig. 2B). Contrasting with this loss of motility, motility persisted and became excessive in Dm*dnah3*^*1*^ mutants (Fig. 2A), with amplification gains around 20 (Fig. 2B). Hence, in addition to affecting electrical auditory neuron responses, Dm*dnai2*^*1*^ abolishes active amplification whereas Dm*dnah3*^*1*^ facilitates this amplification, documenting that auditory neuron motility requires Dm*dnai2* and must be negatively regulated by Dm*dnah3*.

To determine whether these mutant phenotypes arise from the *Mi* insertions, we generated precise excisions by mobilizing the respective *Mi* elements. Both Dm*dnai2*^*ex1*^ and Dm*dnah3*^*ex1*^ revertants displayed normal sound-evoked electrical and mechanical responses that resembled those of the controls (Fig. 2A,B). A full restoration of normal sound-evoked responses was also observed in Dm*dnah3*^*1*^ and Dm*dnai2*^*1*^ mutants when we expressed respective genomic rescue fragments in the respective mutant backgrounds (Fig. 2A,B). In Dm*dnai2*^*1*^ mutants, normal hearing was also restored when we targeted the expression of *UAS-*Dm*dnai2-YFP* to chordotonal neurons via F-Gal4 (Fig. 2A), which expresses Gal4 under the control of the *nan* enhancer/promoter^37^. Accordingly, the diverse auditory phenotypes observed in Dm*dnah3*^*1*^ and Dm*dnai2*^*1*^ mutants can all be ascribed to the mutations in the respective dynein genes.

### *Dm*dnah3 regulates motility together with Nan-Iav TRPV channels

**Figure 3:**
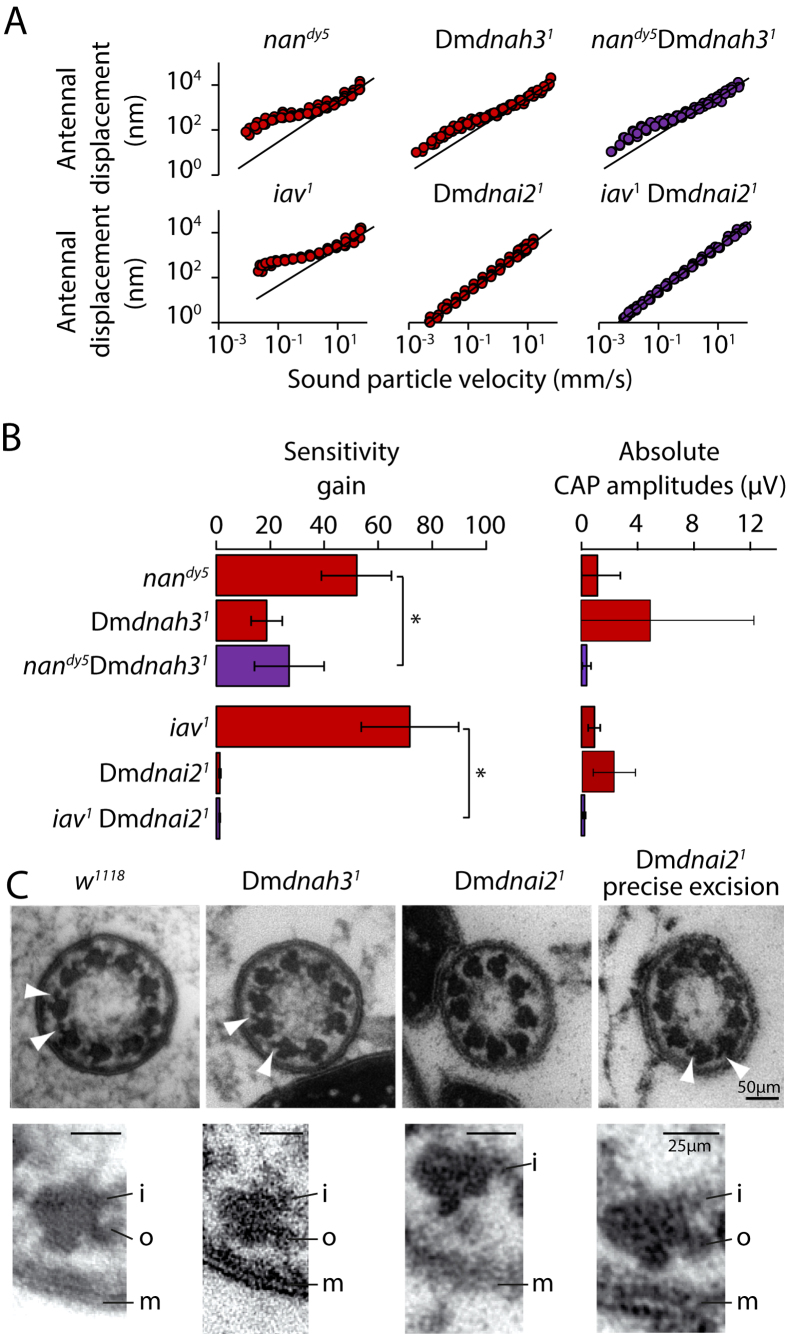
Epistatic relations between axonemal dyneins and Nan-Iav TRP channels and ciliary dynein arm integrity. (**A**) Antennal displacements as functions of the sound particle velocity in single *nan*^*dy5*^ and *iav*^*1*^ mutants (left), single Dm*dnah3*^*1*^ and Dm*dnai2*^*1*^ mutants (middle), and respective double mutants (right). Black lines indicate linearity. Antennal nonlinearity due to auditory neuron motility in the double mutants resembles that of the respective single dynein mutants (data from N = 5 flies/antennae per strain). (**B**) Respective nonlinear amplification gains (means ± SD). *significant difference (p < 0.05) from *w*^*1118*^ controls: (Kruskal-Wallis test followed by two-tailed Mann-Whitney U-tests with Bonferroni correction). (**C**) Transmission electron micrographs of cross-sections through the proximal region of the mechanosensory primary cilia of Johnston’s organ neurons, depicting the dynein-arms (arrowheads, top row). Bottom: zoom-ins depicting single microtubule doublets and their dynein arms. The inner (i) and outer (o) arms are present in *w*^*1118*^ genetic background controls and Dm*dnah3*^*1*^ mutants, but the outer dynein arms are selectively lost in Dm*dnai2*^*1*^ mutants. In the latter mutants, the outer arms are restored upon precise excision of the *Mi* element in Dm*dnai2*. m: ciliary membrane. Scale bars: 50 nm (top) and 25 nm (bottom).

Mechanical hyper-amplification and virtual loss of electrical sound responses, as observed in Dm*dnah3*^*1*^ mutants, also characterizes flies lacking Nan-Iav TRPV channels (Ref. 12,18). To assess whether Dm*dnah3* and Nan-Iav might operate in the same signaling pathway, we generated double mutants carrying Dm*dnah3*^*1*^ along with *nan*^*dy5*^—a *nan* null allele that abolishes both Iav and Nan proteins from auditory neuron cilia^18^. Dm*dnah3*^*1*^*, nan*^*dy5*^ double mutants entirely lacked sound-evoked electrical potentials, same as single Dm*dnah3*^*1*^ and *nan*^*dy5*^ mutants (Fig. 3A). Mechanical amplification due to ciliary motility was excessive in the double mutants, with amplification gains of around 25 (Fig. 3B). This gain was substantially lower than that of single *nan*^*dy5*^ mutants (gains of around 50) but closely resembled that of single Dm*dnah3*^*1*^ mutants (gains of around 20). This establishes an epistatic relation between *Dm*DNAH3 and Nan-Iav, placing *Dm*DNAH3 downstream of Nan-Iav in a regulatory pathway that controls the mechanical amplification gain.

We also generated double mutants carrying Dm*dnai2*^*1*^ and *iav*^*1*^—an *iav* null allele that abolishes Nan and Iav from auditory neuron cilia^18^, same as *nan*^*dy5*^. Like single Dm*dnai2*^*1*^ and *iav*^*1*^ mutants Dm*dnai2*^*1*^, *iav*^*1*^ double mutants lacked sound-evoked nerve responses. Mechanical amplification was excessive in single *iav*^*1*^ mutants, but entirely abolished in Dm*dnai2*^*1*^, *iav*^*1*^ double mutants, as in single Dm*dnai2*^*1*^ mutants (Fig. 3A,B). Hence, *Dm*DNAI2 is also placed downstream of Nan-Iav in the regulatory pathway that controls amplification, analogous to the respective placement of the NOMPC TRP channel^12^, which also operates downstream of Nan-Iav in amplificatory gain control^12^ and is essential for mechanical amplification^13^.

### Axonemal dynein arms in auditory neuron cilia require *Dm*DNAI2

To test whether axonemal dyneins are required for the anatomical integrity of Johnston’s organ neurons, we analyzed their cellular morphologies in Dm*dnah3*^*1*^ and Dm*dnai2*^*1*^ mutant flies. No gross morphological defects were seen when we visualized the neurons with an anti-horseradish peroxidase (HRP) antibody (Fig. S6A), and antibody staining against NOMPC and Iav documented a normal ciliary localization of these TRPs (Fig. S6B). Inspecting the ciliary axonemes with transmission electron microscopy revealed that the dynein arms in the proximal region of the cilium persist and seem structurally uncompromised in Dm*dnah3*^*1*^ mutants, but that the outer arms are selectively lost in Dm*dnai2*^*1*^ mutant flies (Fig. 3C). In the latter mutants, the outer dynein arms were restored upon precise excision of the respective *Mi* element (Fig. 3C), documenting that the outer dynein arms require *Dm*DNAI2.

### *Dm*DNAH3 and *Dm*DNAI2 impair sperm competition

**Figure 4:**
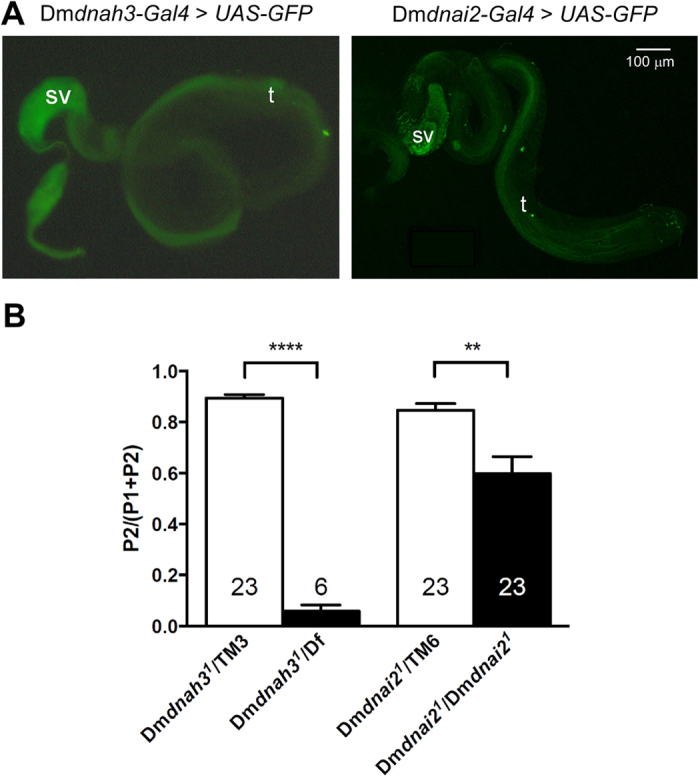
Dm*dnah3* and Dm*dnai2* are expressed in sperm and impair sperm competition. (**A**) Regulatory sequences of Dm*dnah3* (left) and Dm*dnai2* (right) fused to Gal4 can both drive expression of fluorescent markers in sperm. Expression is seen both in developing sperm tails in the testis (t) as well as in mature sperm in the seminal vesicles (sv). (**B**) Mutations in *Dmdnah3* and *Dmdnai2* impair sperm competition. Dm*dnah3*^*1*^and Dm*dnai2*^*1*^ mutant males (black bars) and their heterozygous controls (white bars) were tested for their ability to displace sperm in previously mated females. Sperm displacement was measured as the proportion of offspring from the second male (P2/P1 + P2). Bars represent means, error bars represent SEM. Number of males measured for each genotype is shown in or above the bar. **p < 0.01; ****p < 0.0001 (Kruskal-Wallis test followed by two-tailed Mann-Whitney U tests). Data were included only if at least one offspring from the second male was recovered. This conservative approach was to eliminate any possible effect of the mutations on the ability of males to achieve copulation. The numbers of males that were thus not taken into consideration were 0 for Dm*dnah3*^*1*^/TM3 but 23 for Dm*dnah3*^*1*^/Df, and 1 for Dm*dnai2*^*1*^/TM6 male but 4 for Dm*dnai2*^*1*^/Dm*dnai2*^*1*^.

In *Drosophila*, sperm and auditory chordotonal neurons are the only ciliated cells whose cilia generate motility with axonemal dynein arms. Driving UAS-EGFP via Dm*dnah3-GAL4 or* Dm*dnai2-GAL4* revealed that both Dm*dnah3* and Dm*dnai2* are expressed in sperm (Fig. 4A), corroborating the reported presence of *Dm*DNAH3 in sperm revealed by mass spectrometry^38^. We thus analyzed male fertility in Dm*dnah3*^*1*^ and Dm*dnai2*^*1*^ mutants. Homozygous Dm*dnah3*^*1*^ mutants were behaviorally able to mate, but no offspring were recovered, and testis squashes consistently showed sperm that were not motile. Homozygous Dm*dnai2*^*1*^ males, by contrast, if they achieved mating, produced as many males as heterozygous controls, and testis squashes from all males showed motile sperm. Offspring was also obtained from Dm*dnah3*^*1*^ mutants when the Dm*dnah3*^*1*^ mutation was uncovered by the deficiency *Df(3L)BSC371*, suggesting that the loss of male fertility and sperm motility in the homozygous mutants –but not their hearing defects (Fig. S7)—arise from a secondary mutation. To characterize contributions of *Dm*DNAH3 and *Dm*DNAI2 to the function of sperm, we performed sperm competition assays in which *w*^*1118*^ females were first mated to *w; FRT*^40A, *neo*^ *FRT*^*G13, w*+^ males (P1) and then to balanced Dm*dnah3*^*1*^/*TM3* or Dm*dnai2*^*1*^*/TM6* flies and mutant Dm*dnah3*^*1*^/*Df(3L)BSC371* or Dm*dnai2*^*1*^/Dm*dnai2*^*1*^ males (P2). Scoring the offspring revealed that sperm from control males displaced 80–90% of P1 sperm, but sperm from *Dmdnah3*^*1*^*/Df(3L)BSC371* males were almost completely unable to compete, and *Dmdnah2*^*1*^ homozygotes showed a highly significant reduction in sperm competition (Fig. 4B). This extends the roles of axonemal dyneins in sperm competition^39^ to *Dm*DNAH3 and *Dm*DNAI2 and shows that these two proteins, in addition to their auditory requirements, contribute to sperm function and motility.

## Discussion

Much like vertebrate hair cells, *Drosophila* auditory neurons are motile and mechanically amplify the vibrations they transduce^4^,^10^. Whereas mechanical amplification by hair cells involves prestin molecules and, presumably, myosin motors^40^,^41^, amplification by the fly’s auditory chordotonal neurons is now shown to involve dyneins: according to our results, axonemal dyneins are expressed in *Drosophila* chordotonal neurons and are required for their mechanosensory function and mechanical amplification in the ear. *Dm*DNAH3 controls the mechanical amplification gain together with Nan-Iav TRPV channels, and *Dm*DNAI2 seems to be an important constituent of outer dynein arms in auditory neuron primary cilia that is essential for mechanical amplification. That both *Dm*DNAH3 and *Dm*DNAI2 participate in ciliary motility is supported by mutant defects in sperm competition. This establishes the presence of specific axonemal dynein proteins in insect chordotonal neuron cilia and links mechanical amplification by *Drosophila* auditory neurons to primary cilium motility and axonemal dynein motor components.

Judging from the present and previous^10^,^13^,^16^,^17^ findings, it now seems that mechanical amplification by *Drosophila* auditory neurons might arise from the interplay between the force-gating of NOMPC channels and associated active movements of axonemal dynein motors. If so, NOMPC and axonemal dyneins would have to signal along the cilium because they reside in distinct ciliary compartments^6^,^11^, an indirect interaction that might be mediated by the microtubule axoneme: both NOMPC and axonemal dyneins bind to microtubules^42^,^43^,^44^,^45^ and can be activated by force^16^,^45^. Being mechanically coupled through the axoneme, gating movements of the channels thus might directly trigger motor movements, and vice versa, allowing for the fast channel-motor interactions that are required to explain the cycle-by-cycle amplification of vibrations in the *Drosophila* ear, which operates at frequencies above 100 Hz^10^.

The above scenario seems consistent with an amplification model in which the interplay between motors and force-gated channels drives amplification^10^. According to this model, motor movements power amplification by promoting transducer adaptation, whereby the motors actively reclose the mechanotransduction channels when forcing is maintained^10^. Besides NOMPC, Nan-Iav has been surmised to mediate transduction in JO neurons^26^,^46^, yet recent evidence suggests that Nan-Iav cannot be a mechanotransduction channel because it is not mechanosensitive^19^. Correlates of NOMPC-dependent force-gating and channel adaptation have been observed in the fly’s antennal mechanics^10^,^17^,^47^, suggesting that dyneins might drive amplification by promoting adaptation of NOMPC. We now found that these correlates are entirely abolished from the antennal mechanics of Dm*dnai2*^1^ mutants (Fig. S8), documenting that forces no longer gate the channels, possibly because the channels can no longer adapt and, thus, maintain mechanosensitivity. Recent work has shown that NOMPC requires direct interactions with microtubules for mechanogating^44^. Future work will be needed to test whether NOMPC adaptation requires microtubule-bound dyneins.

The different effects on dynein arm morphology and amplification resulting from loss of the *Dm*DNAI2 and *Dm*DNAH3 proteins are consistent with the different functions of their homologs in other species. The IC2 intermediate chain protein encoded by *Dm*DNAI2 is not a force-generating subunit, but IC2 proteins in *Chlamydomonas* and in humans also function in assembly of the outer arms^32^,^33^,^48^,^49^, which are required for flagellar and ciliary motility. In contrast, monomeric inner arm dynein proteins such as *Dm*DNAH3 are not absolutely required for motility: *Chlamydomonas* mutants lacking one such protein show reduced flagellar beating only under viscous load^31^, consistent with our finding that *Dm*DNAH3 mutant sperm function defects are only revealed in competition with normal sperm flagella. The *Drosophila* motor that includes *Dm*DNAH3 may modulate antennal motility in response to TRPV channel activity, mechanical loading, or both. Candidates for the force-generating proteins that drive auditory motility in *Drosophila* are the outer arm heavy chains encoded by *CG9492* and *Dhc93AB*, which both have been detected in the fly’s auditory organ^22^. Rigorously testing their roles in amplification must involve genetic rescue experiments as presented here for Dm*dnai2* and Dm*dnah3*, and demonstrating that they drive amplification and ion channel adaptation will require targeted manipulations of their active motor properties^50^,^51^.

## Methods

### Nomenclature

A convention to date has been to identify autosomal *Drosophila* dynein heavy chain (Dhc) genes by their chromosomal location. *CG17150*, however, is located in the same cytogenetic interval (64C) as the cytoplasmic dynein gene *Dhc64C*, so following convention in this case could cause confusion between these distinct dynein species.

### Flies

*w*^*1118*^ was used as genetic background control and *Canton-S* as wild-type strain. *w*^*1118*^; *Mi*{*ET1*}*CG17150*^*MB05004*^/*TM6C*, *Sb*^*1*^, *w*^*1118*^; *Df*(*3L*)*BSC371*/*TM6C*, *Sb*^*1*^ *cu*^*1*^, *w*^*1118*^; *Mi*{*ET1*}*CG6053*^*MB06262*^, *SM6a*-*Trans*(*MiT*)*hs24*/*sna*^*Sco*^, *nan*^*dy5*^, *iav*^*1*^, *F*-*GAL4,* and *UAS-GFP-T2*, strains were obtained from Bloomington Drosophila Stock Center. *w; FRT*^40A, *neo*^ *FRT*^*G13, w+*^ were previously described^52^. Flies were raised on standard cornmeal medium under a 12 hr light-dark cycle at 25 °C temperature and 75% humidity. Heat-shock inducible Minos transposase, *SM6a-Trans*(*MiT24*)*hs24*, was used to excise the Minos transposon from *Mi*{*ET1*}*CG17150*^*MB05004*^ and *Mi*{*ET1*}*CG6053*^*MB06262*^ strains. To generate excisions, *SM6a-Trans*(*MiT24*)*hs24* flies were crossed with both *Mi*{*ET1*}*CG17150*^*MB05004*^ and *Mi*{*ET1*}*CG6053*^*MB06262*^ flies and their progeny was heat-shocked daily for one hour at 37 °C during pupariation.

### Promoter fusions and reporter constructs

Genomic DNA was extracted from WT-flies using Qiagen DNeasy Blood and Tissue kit. To generate Dm*dnai2*-GAL4 promoter fusions, the putative promoter gene sequences were amplified using the primers 5′-CGAATTCAAATCAAACCAGCTCTTGTAGTTACC-3′ (forward) and 5′-CGGATCCGAGTTCTCGGTGAACACCACCT-3′ (reverse) and inserted into pPTGAL vector^53^. To generate UAS-Dm*dnai2* rescu*e* constructs, a coding sequence (cds) clone of *CG6053* (IP13643, DGRC, Vienna) was used to amplify the CG6053 cDNA region using the 5′-CCGAATTCAAATATTTTGCTAAGTTTCCGATTGAAATGGAA-3′ (forward) and 5′-GATCTAGACAGCCTCCTCCGCATCCTCTAC-3′ (reverse) primers. The amplicon was inserted into an UAS-attP vector obtained from the Konrad Bassler lab at the University of Zurich. The same construct was tagged with YFP to generate the UAS-Dm*dnai2*-YFP reporters. To generate a genomic rescue construct for Dm*dnai2*, the BACPAC clone pBAC70G22 (P(acman) resource centre, http://www.pacmanfly.org/), which contains a 20 kb region spanning *11683101 to 11705106* of chromosome arm 3L that includes the complete genomic region of *CG6053* (which spans from *11690438* to *11692363*), was inserted into the *w*^*1118*^ background. Microinjections were performed by BestGene Inc (http://www.thebestgene.com/).

### RNA extraction and cDNA preparation

RNA was extracted from whole flies using the ZR Tissue & Insect RNA Microprep Kit (Zymo Research, Irvine, CA) following the manufacturer’s instructions, and cDNA was synthetized using the QuantiTect Reverse Transcription Kit (Qiagen, Venlo, Netherlands).

### Sound responses and correlates of mechanotransduction

The methods to assess sound-evoked antennal displacements and nerve responses as well as mechanical correlates of force-gating and adaptation have been described^17^,^47^,^54^. To compare auditory sensitivities across flies, nerve response amplitudes were plotted against the sound particle velocity and the increasing slope was fitted with a Hill equation. The sound particle velocity corresponding to 10% of the maximum amplitude assumed by the Hill fit was defined as the threshold intensity^22^.

### Immunohistochemistry

The following primary antibodies were used: mouse anti-Futsch (22c10) (Developmental Studies Hybridoma Bank, http://dshb.biology.uiowa.edu/), rabbit anti-GFP (Abcam, Cambridge, UK), anti-IAV (gift from Changsoo Kim, Seoul), rabbit anti-HRP (Invitrogen, Carlsbad, CA), mouse anti-NOMPC (gift from Jonathan Howard, Yale), and Alexa Fluor 647 conjugated anti-phalloidin (Molecular Probes, Eugene, OR). The secondary antibodies used are Alexa Fluor anti-rabbit 488, Alexa Fluor anti-mouse 488, Alexa Fluor anti-mouse 546, and Alexa Fluor anti-rat 633 (Invitrogen).

For antibody staining, fly heads (Johnston’s organ) were washed with 0.1% PBT (0.2% bovine serum albumin, 0.1% Triton-X in phosphate buffered saline) and fixed in 4% paraformaldehyde (PFA) for one hour. Heads were embedded in gelatin-albumin and sectioned at 40 to 50 μm with a vibratome (Leica, Oberkochen, Germany). Expression in the pentascolopidial organ was examined in larval fillets that were fixed for one hour in 4% PFA.

Upon sample preparation, samples were washed three times for 15 minutes in 1% PBT and blocked in 0.25% bovine serum albumin (BSA) and 10% normal goat serum (NGS) for one hour at room temperature. Antibodies (1%) were added to the samples, and were incubated at 4 °C temperature overnight. The samples were washed three times for 5 minutes per wash in PBT and treated with anti-rabbit and -mouse fluorophore conjugated secondary antibodies at a 1:300 concentration in PBS-T for 3 hours at room temperature. After washing the samples three times for 15 minutes, they were mounted in DABCO (Sigma-Aldrich, St. Lewis, MI). Staining was visualized with a Leica SP8 confocal microscope, and images were analyzed with ImageJ.

### Transmission electron microscopy

To maintain the structural integrity of the samples, we used high pressure freezing. A 200 μm deep aluminium specimen carrier (Leica, 16770141 Type A) was filled with 20% Polyvinylpyrrolidone (Sigma-Aldrich) in fly ringer (70 mM NaCl, 5 mM KCl, 10 mM NaHCO_3_, 1,5 mM CaCl_2_ (*2H2O), 4 mM MgCl_2_, 5 mM trehalose, 115 mM saccharose, 5 mM HEPES). Dissected antennae were transferred into the platelet, closed with a second specimen carrier (16770142 Type B) and immediately frozen using a Leica EM HPM 100. Freeze substitution was carried out in a Leica EM AFS at −90 °C for 100 h in 0.1% tannic acid and another 40 h in 2% OsO_4_ (each mass/volume (w/v) in dry acetone + 1% water) while slowly increasing temperature^55^. Antennae were then infiltrated with Durcupan (Fluka, 30% for 3 h, 70% for 3 h, 90% overnight, 2 × 100% for 3 h and thin embedded between two glass slides for 48 h at 70 °C. For microscopic examination, 70 nm sections were cut using a Reichert Ultracut E ultramicrotome and transferred onto Formvar-coated copper mesh grids (PLANO G2405C). Sections were post-stained for 40 min with 4% (w/v) uranyl acetate in water and for 2 min with lead citrate^56^. Micrographs were taken in a JEOL electron microscope (JEM 1011, JEOL, Eching, Germany) with a Gatan Orius 1200A camera (Gatan, Munich, Germany).

### Sperm competition

Females (*w*^*1118*^) were crossed to *w*; *FRT*^*40A*,*neo*^ *FRT*^*G13 w*+^ males in groups of 10 pairs per vial. These first males were removed after 48 hrs. After an additional 48 hrs, the females were crossed individually to second males of the following four genotypes: Dm*dnah3*^*1*^/*TM3* and their sibs Dm*dnah3*^*1*^/*Df(3L)BSC371*, Dm*dnai2*^*1*^*/TM6C* and their sibs Dm*dnai2*^*1*^/Dm*dnai2*^*1*^. After 60 hrs, the second males were discarded and the females were transferred to fresh vials for 5 days, and subsequently discarded as well. All offspring from both vials for each second male were scored for white (P2) or orange (P1) eyes. For each second male, sperm displacement was calculated as P2/(P1 + P2). Statistical significance was determined by Mann-Whitney U tests.

## Additional Information

**How to cite this article**: Karak, S. *et al.* Diverse Roles of Axonemal Dyneins in *Drosophila* Auditory Neuron Function and Mechanical Amplification in Hearing. *Sci. Rep.* **5**, 17085; doi: 10.1038/srep17085 (2015).

## Supplementary Material

Supplementary Information
