# Supplementary material for: Diverse Roles of Axonemal Dyneins in Drosophila Auditory Neuron Function and Mechanical Amplification in Hearing
Source: Sci Rep. 2015 Nov 26;5:17085. doi: 10.1038/srep17085 (PMC4660584; doi:10.1038/srep17085)
Supplement: Supplementary Information [file srep17085-s1.pdf]

Supplementary Information

Diverse Roles of Axonemal Dyneins in *Drosophila* Auditory Neuron Function and Mechanical Amplification in Hearing

Somdatta Karak, Julie S. Jacobs, Maike Kittelmann, Christian Spalthoff, Radoslaw Katana, Elena Sivan-Loukianova, Michael A. Schon, Maurice J. Kernan, Daniel F. Eberl & Martin C. Göpfert

Supplemental Figures

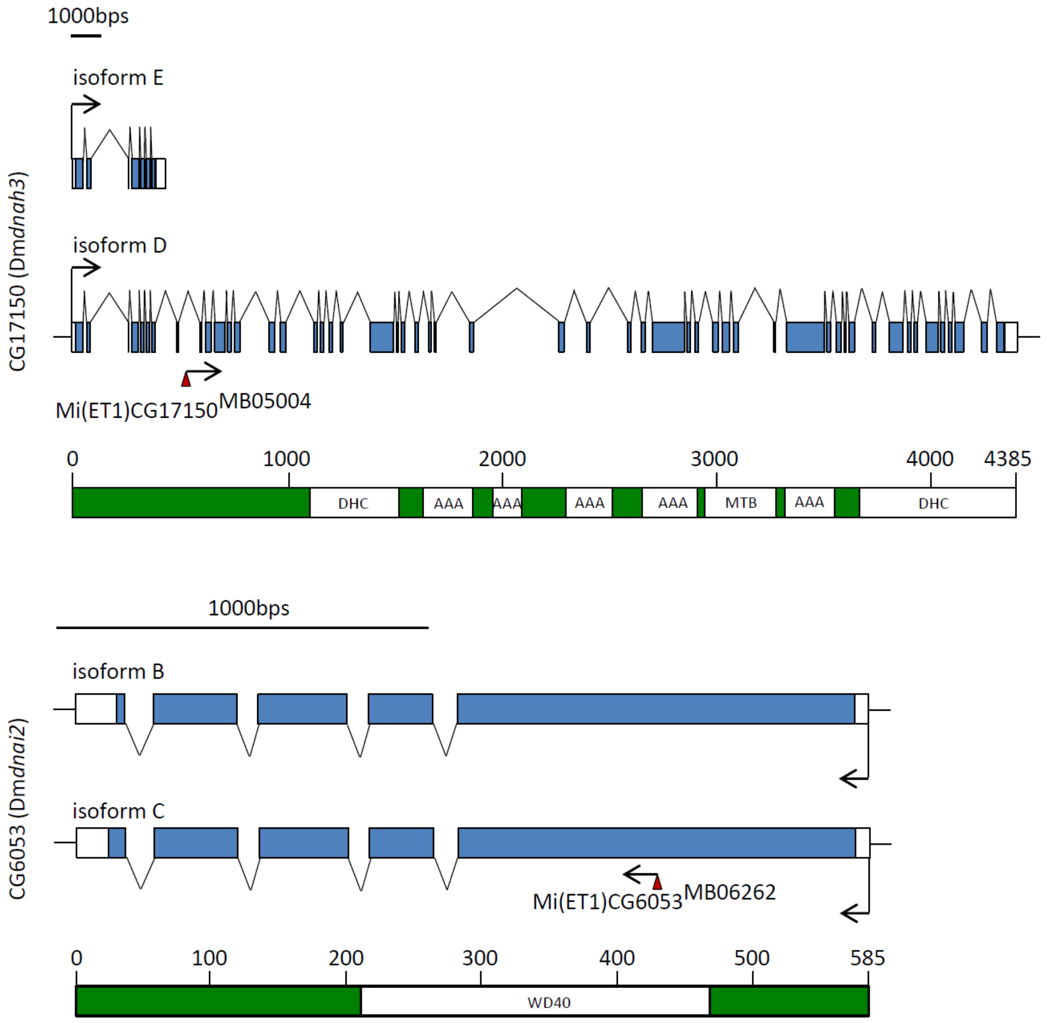

Figure S1. Dm*dnah3* and Dm*dnai2* gene and protein structures, depicting schemata of their isoforms (exons: blue boxes; introns: black lines), Minos insertion sites, and protein structures (green; white boxes: conserved domains). DHC: dynein heavy chain superfamily domain; AAA: ATPase associated with diverse cellular activities; MTB: microtubule binding domain; WD40: WD40 domain, which acts as a site of protein-protein interactions. For Dm*dnah3*, the protein structure is only shown for the long isoform D.

Gene #	Gene name	Class	Protein accession #
Drosophila			
CG6053	*Dnai2*	IC2	NP_648497.2
CG1571		IC2	NP_572435.1
CG10859		IC2	NP_609651.1
CG14838		IC140	NP_648138.1
CG13930		IC138	NP_647645.1
CG7051	*Dic-61B*	IC138	NP_728477.1
CG9313		IC1	NP_611530.5
CG18000	*short wing*	Cdic	NP_477069.1
CG9580	*Sdic1*	Cdic	NP_524670.2
CG33497	*Sdic2*	Cdic	NP_996516.1
CG32823	*Sdic3*	Cdic	NP_728323.3
CG33499	*Sdic4*	Cdic	NP_001285485.2
Chlamydomonas			
DIC1	*ODA9*	IC1	Q39578.3
DIC2	*ODA6*	IC2	P27766.1
DIC3	*IDA7*	IC140	EDP01123.1
DIC4	*BOP5*	IC138	EDP00613.1
	FAP133	D1bIC	AEY82452.1
**Human**			
	DNAI1	IC1	Q9UI46.1
	DNAI2	IC2	Q9GZS0.2
	WDR63	IC140	Q8IWG1.1
	WDR78	IC138	Q5VTH9.1
	DYNC1I1	Cdic	O14576.2
	DYNC1I2	Cdic	Q13409.3
	WDR34	IFT	Q96EX3.2

Figure S2. Names and Genbank identifiers of the intermediate chain protein sequences included in the alignments and trees. Gene nomenclature and classification of the *Chlamydomonas* and human orthologs follows that outlined in Ref. 29. The rapidly evolving *Sdic* genes, found in the *melanogaster* species group only, are the result of a gene fusion and duplications of the cytoplasmic dynein intermediate chain *short wing*/*Cdic* (Ref. S1).

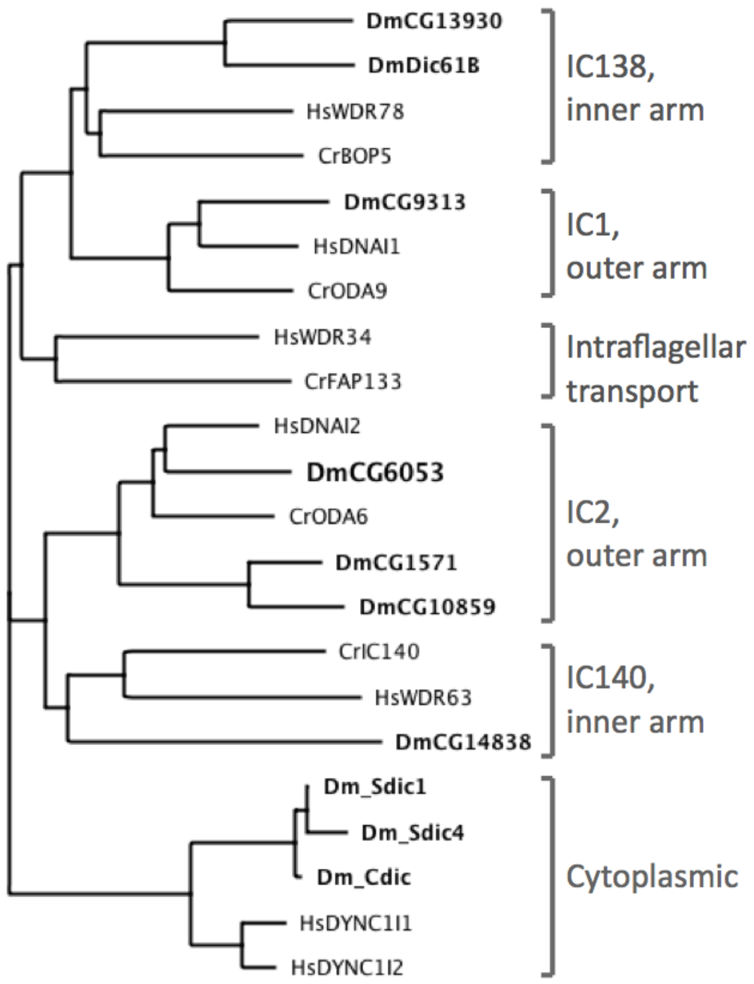

Figure S3. Unrooted similarity tree of the WD-repeat dynein intermediate chain proteins encoded in the *Drosophila* (prefix Dm) *Chlamydomonas* (Cr) and human (Hs) genomes. The tree was generated in Geneious using the neighbor-joining method. Ten of twelve *Drosophila* IC proteins are included; the two omitted, Sdic2 and Sdic3, are from the *Sdic* gene repeat array and are very similar to Sdic1&4. The subgroups are identified following Refs. 28 and 29. Note that the *Sdic* genes, though derived from and similar to the cytoplasmic dynein IC gene *Cdic*, are expressed in sperm (Ref. S1).

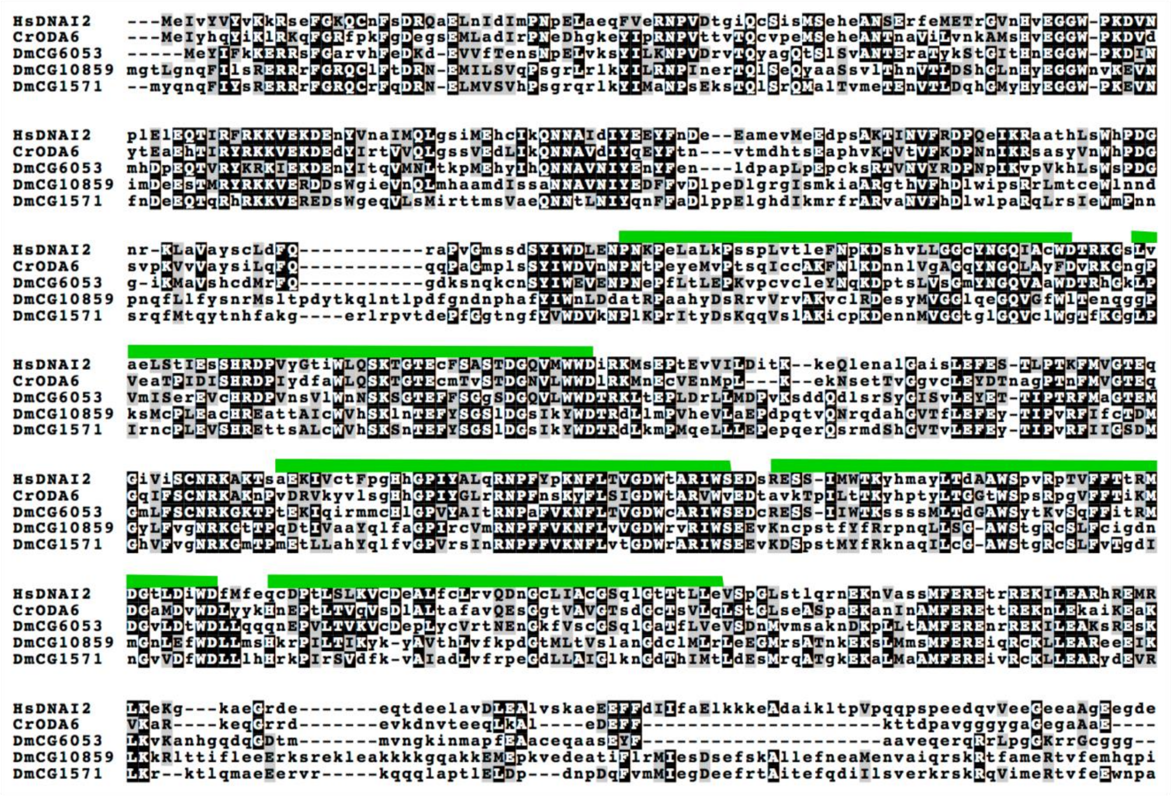

Figure S4. CLUSTALW alignment of the human (HsDNAI2), *Chlamydomonas* (CrODA6), and *Drosophila* (CG6053, CG10859, CG1571) IC2 class dynein intermediate chain proteins. Amino acid identities (black) and similarities (gray) are shaded. Green bars demarcate WD repeats identified in three or more of the sequences.

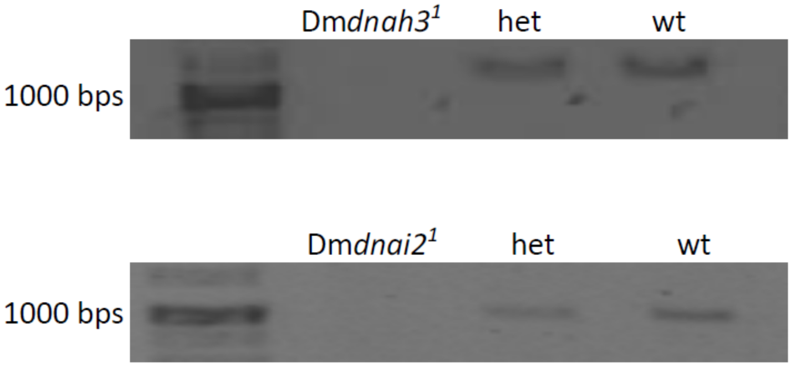

Figure S5. Absence of transcripts in Dm*dnah3*^{1} and Dm*dnai2*^{1} mutants revealed by RT-PCR. RT-PCR was performed using cDNA from homozygous and heterozygous (het) mutants and Canton-S wild-type controls (wt).

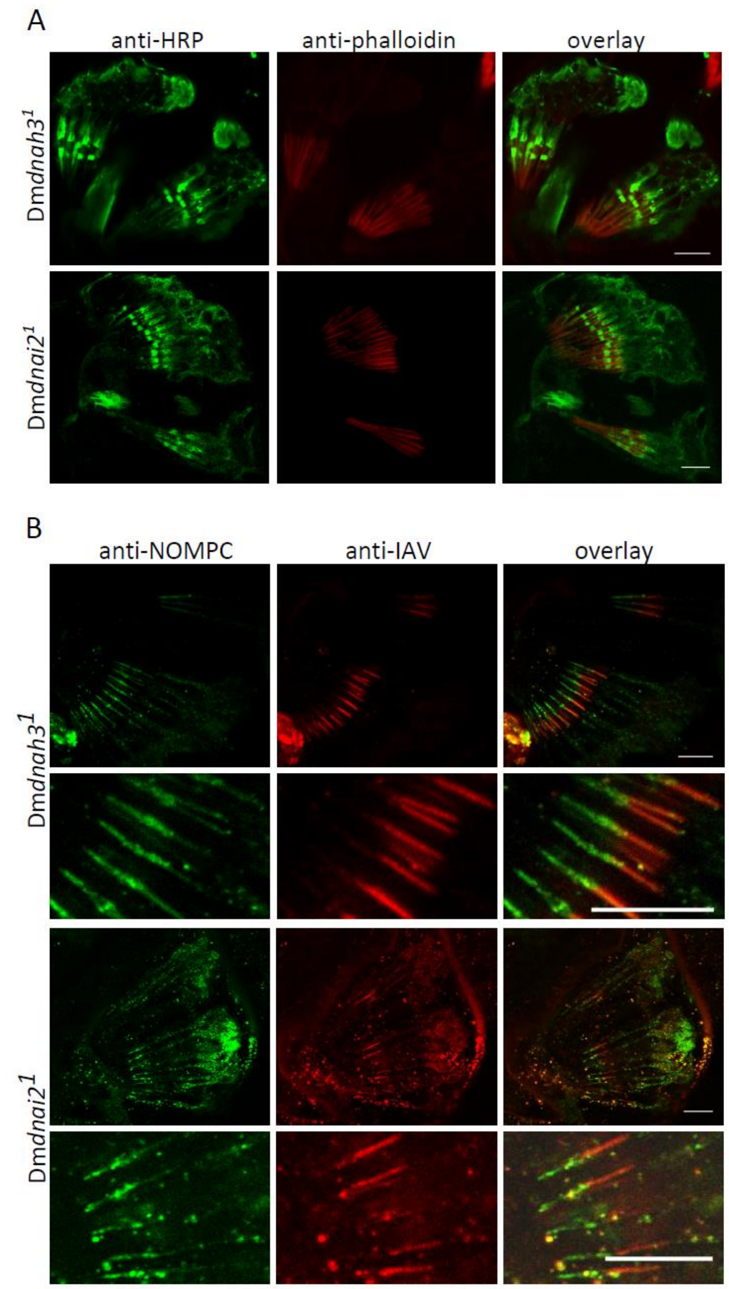

Figure S6. Johnston's organ neuron morphology in Dm*dnah3*^{1} and Dm*dnai2*^{1} mutants and ciliary localization of the ciliary TRP channels NOMPC and Nan-Iav. (**A**) Neuron morphology, revealed staining the neurons with anti-HRP and counterstaining with phalloidin, which recognizes filamentous actin. (**B**) Ciliary localization of NOMPC and Nan-Iav, revealed using anti-NOMPC (Ref. 15) and anti-Iav (Ref. 18) antibodies. Scale bars: 10 μm. Neuron morphologies seem normal and NOMPC and Iav localize properly to the tips and proximal regions of the cilia, respectively.

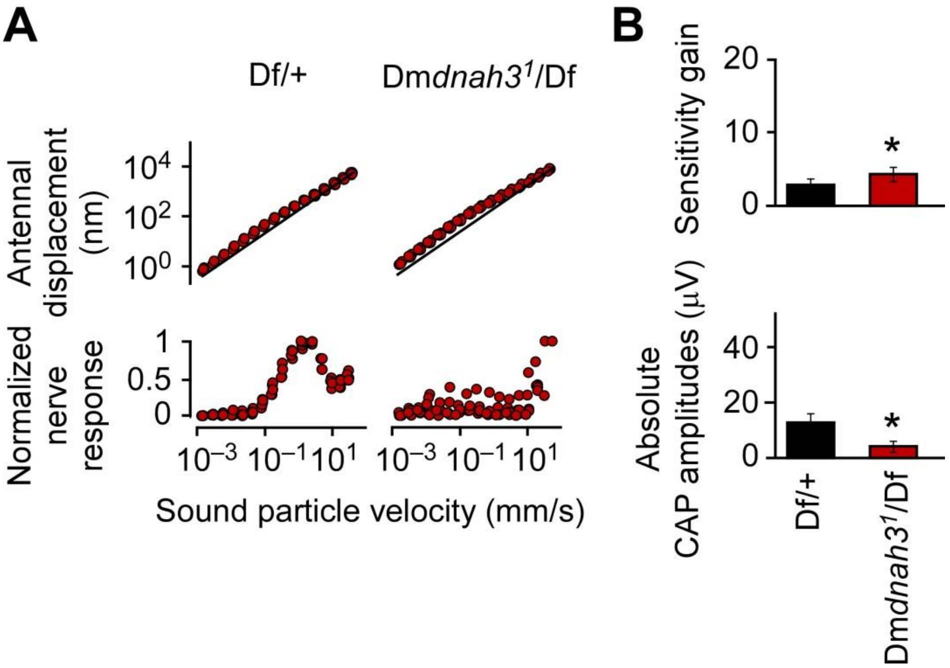

Figure S7. Auditory performance in Dm*dnah3*^{1}/Df mutants, in which the mutation in the dynein is uncovered by the deficiency *Df(3L)BSC371*, compared to heterozygous Df/+ controls, obtained by crossing the deficiency against the genetic background *w*^{1118}. **(A)** Pure tone-evoked antennal displacements (top) and normalized amplitude of the associated nerve response (bottom) as functions of the sound particle velocity (N = 5 flies per strain, for details see Fig. 2). **(B)** Corresponding sensitivity gain due to active amplification by Johnston's organ neurons (top) and maximum amplitudes of the compound action potentials (CAPs) recorded from the antennal nerve (bottom) (means ± SD). *: significant difference from heterozygous controls: ($p < 0.05$, two-tailed Mann-Whitney U-tests). As in homozygous Dm*dnah3*^{1} mutants, amplification persists and is slightly excessive in Dm*dnah3*^{1}/Df mutants, and sound-evoked nerve responses are virtually lost.

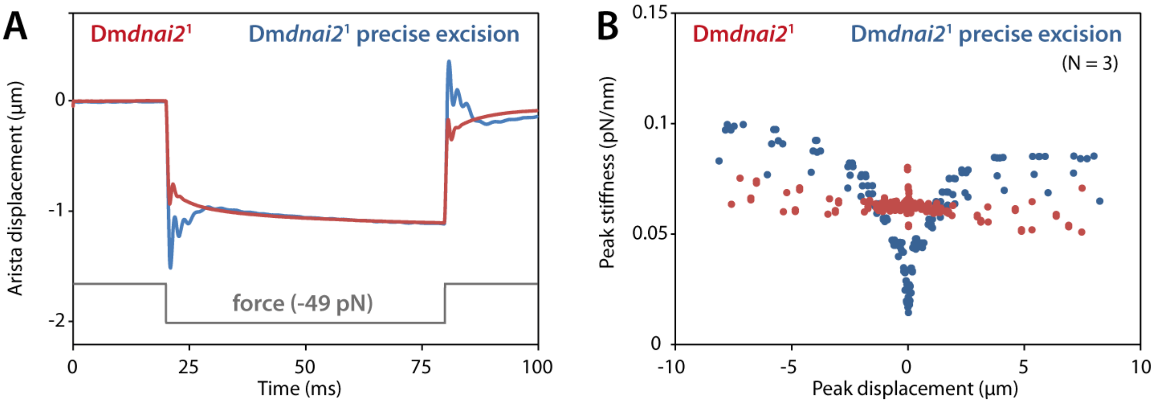

Figure S8. Loss of *Dm*DNAI2 abolishes mechanical correlates of force-gating in the fly's antennal mechanics. **(A)** Displacement response of the antenna measured at the tip of the antennal arista to a -49 pN force step in a Dm*dnai2*^{1} mutant (red trace) and a precise excision control (blue trace). The antenna of the mutant lacks the transient displacement overshoots at the beginning and the end of the force step that reportedly arise from the force-gating of ion channels in Johnston's organ neurons[10,47]. **(B)** Slope stiffness of the antenna at the displacement peak plotted against the corresponding arista displacement. In controls, the force-gating of ion channels introduces a nonlinear gating compliance in the antennal mechanics, rendering the antennal stiffness minimal for zero displacement (and zero force). This nonlinear gating compliance is abolished in Dm*dnai2*^{1} mutants, signaling that the responsible channels no more gate in response to force (pooled data from N = 3 flies each).

Supplementary references

Ref. S1 Ponce, R. & Hartl, D.L. The evolution of the novel Sdic gene cluster in *Drosophila*. *Gene* **376**, 174–183 (2006).
